# Association between oral 5-ASA adherence and health care utilization and costs among patients with active ulcerative colitis

**DOI:** 10.1186/1471-230X-12-132

**Published:** 2012-09-24

**Authors:** Debanjali Mitra, Paul Hodgkins, Linnette Yen, Keith L Davis, Russell D Cohen

**Affiliations:** 1 RTI Health Solutions, 3040 Cornwallis Road, Post Office Box 12194, Research Triangle Park, NC, 27709-2194, USA; 2 Shire Development Inc, 725 Chesterbrook Boulevard, Chesterbrook, PA, 19087, USA; 3 The University of Chicago Medical Center, 5841 South Maryland Avenue, Chicago, IL, 60637, USA

## Abstract

**Background:**

Observational cohort study to assess the association between adherence to oral 5-aminosalicylates (5-ASAs) and all-cause costs and health care utilization among patients with active ulcerative colitis (UC) in the United States.

**Methods:**

Retrospective analysis of insurance claims from June 1997 to August 2006 in the LifeLink Database. Patient criteria: aged 18 or older with one or more claim(s) between June 1997 and August 2005 for a UC diagnosis and at least one oral 5-ASA prescription on or after the first observed UC diagnosis; continuous enrollment for at least 6 months prior to and 12 months following 5-ASA initiation (index date). As a proxy for active disease, patients needed to have at least two UC-specific non-pharmacy claims, at least 30 days of 5-ASA treatment and at least one corticosteroid prescription within the 12-month post-index period. Cumulative exposure to oral 5-ASAs over the 12-month period was calculated using the medication possession ratio (MPR). Patients with an MPR of at least 0.80 were classified as adherent. All-cause medical and pharmacy resource utilization and costs were computed over the 12-month post-index period and compared between adherent and nonadherent patients.

**Results:**

1,693 UC patients met study inclusion criteria: 72% were nonadherent to 5-ASA treatment (n = 1,217) and 28% were adherent (n = 476) in the 12-month study period. Compared with nonadherent patients, adherent patients had 31% fewer hospitalizations (*P* = 0.0025) and 34% fewer emergency department admissions (*P* = 0.0016). Adherent patients had 25% more pharmacy prescriptions overall (*P* <0.0001) and 71% more UC-related pharmacy prescriptions (*P* <0.0001) than did nonadherent patients. Total all-cause health care utilization was 1.13 times higher for adherent patients than for nonadherent patients (*P* = 0.0002). After adjusting for covariates, total all-cause costs were 29% higher for nonadherent patients than for adherent patients (mean [95% confidence interval]: $13,465 [$13,094, $13,835] vs $17,339 [$17,033, $17,645]).

**Conclusions:**

Approximately three-quarters of patients with active UC were not adherent with their prescribed doses of oral 5-ASA. Nonadherence was associated with higher total all-cause costs. The key driver of decreased costs among adherent patients was inpatient hospitalizations, which more than offset these patients’ expected higher pharmacy costs.

## Background

Ulcerative colitis (UC) is a chronic inflammatory bowel disease characterized by mucosal inflammation in the colon. Usually, UC affects individuals between 15 and 40 years of age, although it can be found among patients of any age. The annual incidence rate of UC in the United States (US) is estimated to be eight cases per 100,000 individuals[1]. UC is an expensive medical condition, imposing a significant burden on patients, employers, and third-party payers. In 1998, the total direct cost of UC in the US was estimated to be $388 million, with pharmacotherapy contributing to $138 million of these costs and inpatient hospitalizations accounting for 50% of the total expenditure [2]. The reported annual direct economic burden per patient varies significantly in the literature. Kappleman et al., [3] using an administrative claims data set, found the mean annual direct health care cost of UC to be $5,066 per patient per year (in 2004 US dollars); 38% of costs were attributable to hospitalization, 35% to outpatient care, and 27% to pharmaceutical claims. Another study found the mean annual all-cause total health care costs for patients with UC to be $13,233 (in 2005 US dollars), 44% of which consisted of inpatient hospitalization costs [4].

Because the underlying cause is usually unclear, treatment of UC is typically aimed at first controlling inflammation and symptoms (i.e., inducing remission) and then maintaining that control (i.e., maintaining remission) [5,6]. According to the American College of Gastroenterology practice guidelines for adult UC patients, [7] oral therapy with 5-aminosalicylic acids (5-ASAs) such as mesalamine, balsalazide, sulfasalazine, and olsalazine is typically used as first-line therapy for mild to moderate UC. Therapy with 5-ASAs has been shown to be efficacious and well tolerated both for the treatment of active disease [6] and for the maintenance of remission [5]. However, some of the current oral formulations of 5-ASAs are inconvenient for patients because patients require multiple doses per day and/or multiple tablets per dose. In a chronic disease such as UC, where patients may be on long-term or lifelong medication support, such treatment imposes a significant burden on UC patients, and potentially can reduce quality of life and can negatively impact adherence to the treatment. Previous studies have found that, for the patient being treated for a chronic condition, nonadherence to the treatment can cause a reduction in treatment benefits, [8] can bias clinician assessment of the treatment’s effectiveness, [9] and can lead to poorer disease prognosis [10]. Further, nonadherence to treatment for chronic conditions has been linked to increased health care utilization [11].

Previous studies have demonstrated that adherence and persistence with 5-ASAs in patients with UC is usually low. In a study using the IMS LifeLink™ database (IMS Health Incorporated, Danbury, Connecticut; formerly PHARMetrics® Inc.), Yen et al. [12] found that only 15.2% of patients were adherent to oral 5-ASA therapy in the first year following treatment initiation. Another study found that among patients receiving pharmacotherapy for gastrointestinal conditions, only 22% were persistent with their oral 5-ASA therapy [13]. In a review article on adherence to UC therapy, Kane[14] concluded that failure to adhere to the prescribed UC regimen is associated with an increased risk of symptomatic relapse, greater risk of disease progression (e.g., development of colorectal cancer), decreased quality of life, and higher overall cost of care. Improving medication adherence in UC patients therefore could lead to reduced health care costs and improved outcomes.

Although the significant economic burden of UC and the low rate of adherence with UC medications has been well established in the literature, the impact of adherence with oral 5-ASA therapies on health care utilization and associated costs among patients with active UC has not been widely investigated. In this study, we analyzed administrative claims data from a large managed care population in the US to examine the impact of oral 5-ASA adherence on all-cause health care utilization and costs (both medical and pharmacy) among adult patients with evidence of active UC.

## Methods

### Data source

Data were extracted from the IMS LifeLink database. At the time of our study, the database included enrollment, medical, and prescription information from 75 managed care health plans covering more than 40 million unique patients and over 2 billion health care transactions in the US from 1997 to 2006. The LifeLink database comprises longitudinal insurance claims from managed care organizations in all four US geographic regions and has an age and gender distribution representative of national managed care enrollment [15]. 

The LifeLink database consists of patient demographics, institutional and professional medical claims for inpatient and outpatient services, outpatient prescription drug claims, and periods of health plan enrollment (start and stop dates). Each medical claim includes date and place of service (e.g., inpatient, outpatient, emergency department [ER]), four International Classification of Diseases, 9th Revision, Clinical Modification (ICD-9-CM) diagnosis codes, and cost data in the form of managed care reimbursements (actual paid amount) for medical services and prescription drugs utilized. Medical claims in the database also include Health Care Financing Administration Common Procedure Coding System and Current Procedural Terminology Version 4 codes indicating the type of procedure (if any) provided, as well as information on provider specialty. Prescription drug claims include the National Drug Code, brand and generic name, quantity dispensed, days’ supply, strength, and paid amount for prescriptions obtained through outpatient or retail pharmacies. Data are tracked longitudinally within patients via de-identified and unique patient numbers.

Due to the de-identified nature of the LifeLink database and the fact that no new data were gathered, our study was granted exemption from the Institutional Review Board at RTI International, which holds a Federal-Wide Assurance (#3331) from the US Department of Health and Human Services’ Office for Human Research Protections; RTI Health Solutions, the organization that conducted this study, is a business unit of RTI International.

### Study population

Our study sample was defined by selecting patients from the LifeLink database (1997–2006) who had any primary or nonprimary diagnosis of UC (ICD-9-CM 556.xx) between June 1997 and August 2005 and at least one prescription claim for an oral 5-ASA on or after the date of each patient’s first observed UC diagnosis. For each patient identified, an index date was defined as the date of the first observed prescription for an oral 5-ASA. To ensure that each patient’s index date was a reasonable marker for oral 5-ASA therapy initiation (or re-initiation after an extended break), patients included in the study sample were required to have at least 6 months of continuous health plan enrollment prior to their index date, with no evidence of 5-ASA use during that time frame. Similarly, to ensure that any observed lack of health care events following 5-ASA therapy initiation was due to a true lack of medical activity and not cessation of insurance, patients included in the study sample were required to have at least 12 months of medical and pharmacy benefits eligibility following their index date. Each patient also was required to have a minimum duration of 30 days of 5-ASA therapy during the 12-month follow-up period. Finally, patients were required to have at least one period of active disease during follow-up, which was identified by proxy as evidence of of at least two UC-specific non-pharmacy claims and at least one corticosteroid prescription within the 12-month post-index date period. Patients with Crohn’s disease (ICD-9-CM 555.xx) were excluded from the study.

### Study measures

#### Baseline patient characteristics

Patient characteristics that were measured at the index date included age, gender, geographic region, insurance payer type (e.g., commercial, self, government sponsored), and health plan type (e.g., health maintenance organization, preferred provider organization). To assess overall comorbidity burden prior to 5-ASA initiation, a Charlson Comorbidity Index (CCI) score, with Deyo adaptation for claims data, was calculated for each patient [16,17]. The CCI includes 17 categories of comorbidities, as defined by ICD-9-CM diagnosis codes, with associated weights corresponding to the severity of the comorbid condition of interest. A higher CCI score represents a higher overall comorbidity burden. A single CCI score was calculated for each patient on the basis of the presence of the corresponding diagnosis during the 6-month period prior to the index date.

#### Adherence measure

Oral 5-ASA adherence was measured using the medication possession ratio (MPR). A systematic literature review [18] found the MPR to be the most widely adopted measure (57% of all studies) in published claims-based analyses of medication adherence. MPR is defined as the proportion of days within an observation period covered by the total days’ supply for a particular study drug within the observation period. The observation period used in the MPR calculation can be either a fixed number of days within a follow-up period (i.e., 365 days following treatment initiation) or the number of days between the first dispense date and end of the days’ supply of the last refill for the study therapy of interest [18]. Oral 5-ASAs are intended to be used continuously and long-term (i.e., chronically) in order to maintain remission. Therefore, adherence for these medications must be evaluated with respect to drug exposure or supply coverage over a longer, fixed period. In this context, early discontinuation would count against MPR, and the use of a denominator that is defined as days between the first dispense and last prescription dates does not capture the negative adherence effect of complete therapy discontinuation.

To account for discontinuation, we defined the denominator of the MPR formula as a fixed value of 365 days (i.e., total days in the fixed, post-index follow-up period) so that those patients who discontinued 5-ASA therapy completely before the end of the follow-up period would correctly have a lower MPR. The following formula was used:

(1) MPR=Total oral 5-ASA days supplied in 12-month follow-up period÷365 days.

Note that changes in medication dose had no impact on how refill adherence was calculated. A patient who had a dose titration upward or downward would have the same adherence as a patient who remained on the same dose, as long as both patients had identical prescription refill patterns. Mean MPR and adherence rates were calculated for the overall 5-ASA class, not separately by individual 5-ASA medications. For example, if a patient switched from mesalamine to sulfasalazine, the patient’s adherence was measured as cumulative exposure to both 5-ASA drugs. Switching between two oral 5-ASA classes therefore did not impact the calculation of MPR.

Patients with an MPR of less than 0.8 (i.e., less than 80% adherence) were classified as nonadherent. The 80% threshold for identifying adherence and nonadherence to chronic-use medications has been previously validated [19] and has been used in numerous previously conducted adherence studies [20-22]. To assess the distribution of MPR among nonadherent patients, patients with an MPR of less than 0.8 were further stratified into four groups: MPR 0.0 to 0.19, 0.20 to 0.39, 0.40 to 0.59, and 0.60 to 0.79.

#### All-cause health care utilization and costs

Total health care services utilized and associated costs during the 12-month period following initiation of 5-ASA therapy were calculated. Costs were calculated from the perspective of the third-party payer and represented actual reimbursed amounts for each service rendered. Our cost data therefore did not take into account patient copayments, co-insurance, and other out-of-pocket expenses. All-cause health care utilization and costs were compared between the group of patients who were adherent to 5-ASA therapy and the group of patients who were not. Differences in baseline demographic characteristics (age, gender, geographic region, health plan type, and payer type) and comorbidity burden (CCI scores) were controlled for when calculating total adjusted all-cause health care utilization. We did not adjust for baseline, i.e., pre-index, health care costs. The adjusted all-cause health care costs were stratified by the following cost sectors: inpatient admissions, ER visits, outpatient office visits, other non-office outpatient/ ancillary visits (e.g., day procedures), and prescription drugs (UC related and non-UC related). UC-related prescriptions were defined as all prescriptions for oral and rectal 5-ASAs, corticosteroids, biologics (infliximab), immunosuppressants, and select antibiotics (ciprofloxacin and metronidazole). All cost estimates were adjusted to 2010 US dollars, using the medical care component of the US Consumer Price Index [23].

### Statistical analyses

All analyses were carried out using SAS (Version 9.1.3) statistical software package (SAS Institute, Inc, Cary, North Carolina). Descriptive analyses entailed the tabular display of the mean value, standard deviation, median, and range of continuous variables of interest and the frequency distribution of categorical variables of interest. For descriptive analyses, chi-squared tests for dichotomous variables and paired t-tests for continuous variables were used to assess statistical differences in the outcomes of interest across all patients, stratified by adherence status.

Multivariable regression analyses were performed to assess differences in all-cause health care utilization and costs between 5-ASA adherent and nonadherent UC patients after controlling for patient demographic characteristics and baseline comorbidity burden. The type of multivariable model estimated depended on the nature of the outcome that was assessed. For count data, outcomes such as number of inpatient admissions and outpatient office visits were used. Poisson regression models were used to compare the rate of resource utilization between the two cohorts. These models provided incidence rate ratios (IRRs), which were obtained by exponentiating the coefficients from each model estimated. The magnitude of the IRR describes the incidence rate of utilization (e.g., number of hospitalizations) for patients adherent to 5-ASA therapy as a multiple of the utilization incidence rate among nonadherent patients. For example, an estimated IRR of 0.5 for a binary covariate defining nonadherence (i.e., 1 = adherent, 0 = nonadherent) suggests that patients who were adherent with 5-ASA therapy had 50% fewer hospitalizations than patients who were nonadherent to 5-ASA therapy.

To assess differences in health care costs between the two study cohorts, a multivariable generalized linear model (GLM) framework was used, with a log-link function and a gamma distribution for the error term to resolve the issue of skewed cost distribution often reported in health care cost data. In comparison with ordinary linear regression involving log-transformed cost data, the GLM methodology offers several advantages, primarily because it estimates covariate-adjusted predicted mean costs on a dollar scale, which can then be compared using Student’s t-test [24-26]. The GLM methodology avoids potential biases resulting from the Duan smearing method for retransforming the predicted coefficient from log-transformed cost data [26-28].

## Results

**Figure 1:**
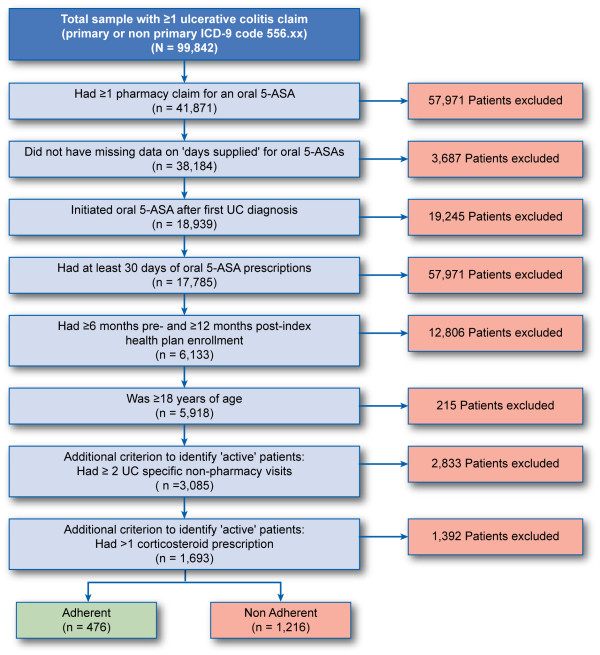
**Sample attrition.** 5-ASA = 5-aminosalicylate; ICD-9-CM = International Classification of Diseases, 9th Revision, Clinical Modification; UC = ulcerative colitis.

We identified 99,842 patients in the LifeLink database who had at least one primary or nonprimary diagnosis code for UC (ICD-9-CM 556.xx) between June 1997 and August 2005. Nearly 60% of these patients (n = 57,971) did not have any pharmacy claims for oral 5-ASA therapy and therefore were excluded. The final study sample consisted of 1,693 patients who met all study inclusion and exclusion criteria. Figure 1 provides a detailed breakdown of the sample attrition.

### Demographic characteristics

Table 1 presents descriptive statistics on various baseline demographic characteristics of the study sample, overall and stratified by the adherence status. The mean age was 42 years for the overall study sample, and there was equal distribution of male and female patients. Nearly 45% of patients resided in the Midwest, with the remaining patients equally distributed between the East, South, and West. Preferred provider organizations and health maintenance organizations were the most common types of health plans, covering 40% and 37% of patients, respectively. The mean CCI score for the overall study sample was 1.23.

**Table 1:** Characteristics of the study population, by 5-ASA adherence status

**Characteristic**	**All 5-ASA Initiators**		**Adherence Status**				***P *Value**^**a**^
			**Not Adherent**		**Adherent**		
	**n**	**%**	**n**	**%**	**n**	**%**	
n (row%)	1693	100.00	1217	100.00	476	100.00	
Gender							
Female	840	49.62	628	51.60	212	44.54	0.0090
Male	853	50.38	589	48.40	264	55.46	—
Age							
Mean (SD)	42.3 (12.8)		42.4 (12.8)		42.2 (12.9)		0.7675
Median	42		42		43.5		
Range (minimum, maximum)	(18, 93)		(18, 93)		(18, 78)		
Distribution (years)							
18-24	165	9.75	112	9.20	53	11.13	0.2283
25-44	775	45.78	576	47.33	199	41.81	0.0403
45-54	433	25.58	304	24.98	129	27.10	0.3684
55-64	280	16.54	191	15.69	89	18.70	0.1349
65+	40	2.36	34	2.79	6	1.26	0.0619
Geographic region							
East	354	20.91	260	21.36	94	19.75	0.4623
Midwest	759	44.83	528	43.39	231	48.53	0.0113
South	306	18.07	238	19.56	68	14.29	0.0557
West	274	16.18	191	15.69	83	17.44	0.3815
Health plan type							
HMO	629	37.15	442	36.32	187	39.29	0.2561
Indemnity	39	2.30	30	2.47	9	1.89	0.4788
POS	261	15.42	181	14.87	80	16.81	0.3218
PPO	681	40.22	505	41.50	176	36.97	0.0881
Unknown	30	1.77	21	1.73	9	1.89	0.8168
Multiple types	53	3.13	38	3.12	15	3.15	0.9756
Payer type							
Commercial	1423	84.05	1020	83.81	403	84.66	0.6672
Medicaid	18	1.06	15	1.23	3	0.63	0.2774
Medicare	48	2.84	40	3.29	8	1.68	0.0735
Self-insured	56	3.31	37	3.04	19	3.99	0.3251
Medigap	2	0.12	2	0.16	0	0.00	0.3762
Unknown	144	8.51	101	8.30	43	9.03	0.6262
Multiple types	2	0.12	2	0.16	0	0.00	0.3762
CCI score							
Mean (SD)	1.23 (1.60)		1.18 (1.59)		1.36 (1.62)		0.0378
Median	0		0		0		
Range (minimum, maximum)	(0, 9)		(0, 9)		(0, 8)		

5-ASA = 5-aminosalicylate; CCI = Charlson Comorbidity Index; HMO = health maintenance organization; POS = point of service; PPO = preferred provider organization; SD = standard deviation.

^a^Chi-square tests for categorical variables, t-tests for continuous variables.

The majority of UC patients (72%) were nonadherent to oral 5-ASA therapy (n = 1,217) during the 12-month study period. Adherent patients were more likely to be male than were nonadherent patients (55% vs 48%; *P* = 0.0090), with no difference in average age between the two groups. A slightly larger percentage of patients in the adherent group resided in the Midwest than did the nonadherent group (49% vs 43%; *P* = 0.0113). Further, adherent patients had a greater baseline comorbidity than did non-adherent patients (CCI score 1.36 vs 1.18; *P* = 0.0378).

### Adherence measure

**Figure 2:**
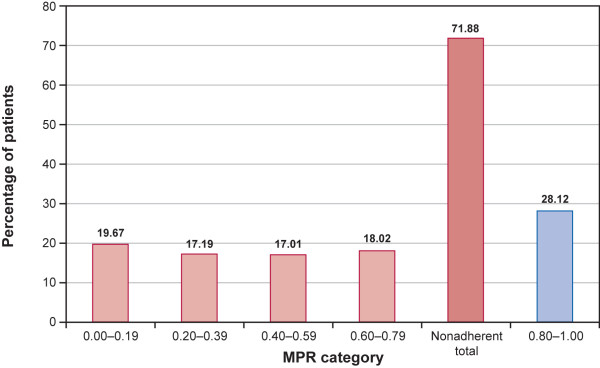
**MPR distribution for overall oral 5-ASA therapy.** 5-ASA = 5-aminosalicylate; MPR = medication possession ratio.

Figure 2 presents the overall MPR distribution for oral 5-ASA therapy. Twenty-eight percent of the sample was adherent to therapy over the 12-month follow-up period, while 20% of patients had an MPR less than 0.20. The mean MPR for the overall study sample was 0.54 (range: 0.08-1.00; median = 0.56; data not shown). Among the adherent group, the mean MPR was 0.90 (range: 0.80-1.00; median = 0.90); and among the nonadherent group, the mean MPR was 0.40 (range: 0.08-0.79; median = 0.38).

### Adjusted health care utilization and costs

Table 2 presents covariate-adjusted IRRs and corresponding 95% confidence intervals (CIs) from the Poisson regression models. The models were used to assess differences in the rate of all-cause health care resource utilization between the adherent and nonadherent patient cohorts. Adherent patients had approximately 31% (IRR = 0.6928; *P* = 0.0025) fewer hospitalizations and 34% (IRR = 0.6584; *P* = 0.0016) fewer ER admissions than did nonadherent patients. Patients who were adherent had 25% more pharmacy prescriptions overall. Adherent patients also had 71% more UC-related pharmacy prescriptions than did nonadherent patients, but the number of non–UC-related prescriptions did not differ between the two groups. The total number of all-cause health care visits across all cost sectors (including inpatient, ER, outpatient, and pharmacy) was 1.13 times higher for adherent patients than for nonadherent patients (IRR = 1.1294; *P* = 0.0002).

**Table 2:** Association between 5-ASA adherence and all-cause health care utilization during 12-month period following 5-ASA initiation

**Dependant Variable**	**Incident Rate Ratio**^**a**^	**Confidence Interval**		***P *Value**
Number of inpatient admissions	0.6928	0.5461	0.8789	0.0025
Number of ER visits	0.6584	0.5077	0.8538	0.0016
Number of outpatient office visits	1.0004	0.9200	1.0878	0.9925
Number of other outpatient/ ancillary visits	0.9306	0.8587	1.0086	0.0798
Number of pharmacy prescriptions	1.2503	1.1624	1.3448	< .0001
Number of UC-related pharmacy prescriptions	1.7095	1.6258	1.7974	< .0001
Number of non–UC-related pharmacy prescriptions	0.9989	0.8862	1.1258	0.9852
Number of non-pharmacy visits	0.9605	0.8937	1.0322	0.2725
Number of total visits	1.1294	1.0595	1.2039	0.0002

5-ASA = 5-aminosalicylate; ER = emergency department; UC = ulcerative colitis.

^a^Incident rate ratios are based on Poisson regression models and show utilization among adherent patients compared to nonadherent patients. All models control for age, gender, geographic region, health plan type, payer type, and Charlson Comorbidity Index scores.

Table 3 presents covariate adjusted predicted mean all-cause health care costs for adherent and nonadherent patients using GLM models. After adjusting for baseline demographic characteristics and the CCI score, the predicted mean all-cause cost per patient for hospitalizations was approximately two times higher for nonadherent patients than it was for adherent patients (mean [95% CI]: $14,542 [$14,314, $14,770] vs $28,727 [$28,443, $29,010]). The mean total cost of ER visits per patient was approximately 28% higher in the nonadherent group than in the adherent group. The mean costs of UC-related pharmacy prescriptions were more than two times higher for patients who were adherent than for those who were not (mean [95% CI]: $3,569 [$3,520, $3,617] vs $1,718 [$1,703, $1,734]). Total all-cause health care costs, including all cost sectors, were about 29% higher for nonadherent patients than for adherent patients (mean [95% CI]: $17,339 [$17,033, $17,645] vs. $13,465 [$13,094, $13,835]). It must be noted that although GLM is a widely used tool to estimated adjusted costs, the predicted cost estimates for any particular cost sector were based on patients with at least one visit in that sector. For example, the mean predicted inpatient costs for the patient cohort were calculated among those patients who had an inpatient visit. This resulted in the mean inpatient costs for patients being much higher than the overall costs for all visits.

**Table 3:** Adjusted health care costs during 12-month period following 5-ASA initiation, by 5-ASA adherence status

**Cost Outcome**	**5-ASA Adherence Status**				
	**Adherent**		**Not Adherent**		
	**Mean Predicted Cost**^**a**^	**95% Confidence Interval**	**Mean Predicted Cost**^**a**^	**95% Confidence Interval**	***P *Value**
Cost of inpatient admissions	$14,541.65	[$14,313.80, $14,769.49]	$28,726.65	[$28,443.11, $29,010.19]	<0.0001
Cost of ER visits	$495.33	[$477.91, $512.76]	$635.95	[$621.95, $649.94]	<0.0001
Cost of outpatient office visits	$1,081.83	[$1,046.79, $1,116.87]	$1,145.67	[$1,121.99, $1,169.35]	0.0031
Cost of other outpatient/ ancillary visits	$3,702.39	[$3,602.08, $3,802.70]	$4,923.29	[$4,837.67, $5,008.91]	<0.0001
Cost of pharmacy prescriptions	$5,309.84	[$5,180.82, $5,438.86]	$3,189.88	[$3,142.40, $3,237.35]	<0.0001
Cost of UC-related pharmacy prescriptions	$3,568.57	[$3,519.87, $3,617.27]	$1,718.20	[$1,702.68, $1,733.73]	<0.0001
Cost of non–UC-related pharmacy prescriptions	$1,845.51	[$1,746.93, $1,944.09]	$1,541.60	[$1,487.04, $1,596.17]	<0.0001
Cost of non-pharmacy visits	$8,101.24	[$7,848.17, $8,354.30]	$14,226.32	[$13,936.79, $14,515.85]	<0.0001
Cost of total visits	$13,464.89	[$13,094.43, $13,835.35]	$17,339.36	[$17,033.28, $17,645.45]	<0.0001

5-ASA = 5-aminosalicylate; ER = emergency department; UC = ulcerative colitis.

^a^Predicted costs are based on generalized linear models and control for age, gender, geographic region, health plan type, payer type, and Charlson Comorbidity Index scores. Separate models were run for each cost sector; therefore, the mean predicted cost for each sector does not sum up to the total predicted cost.

## Discussion

In this study, we examined the association between adherence to 5-ASA therapy and overall health care utilization and costs among patients with active UC. We estimated that only 28% of the UC patients on 5-ASAs who were included in the study were adherent to treatment. This finding was consistent with previous studies that have found adherence with 5-ASAs to be fairly low (range: 15%[12] to 22%[13]). We also found that adherence to oral 5-ASAs generally appeared to be associated with decreased health care utilization and costs, especially the rate of inpatient and ER admissions, despite the fact that adherent patients, on average, had higher baseline CCI scores. One might expect that patients with higher baseline CCI scores would be more likely to have higher medical utilization and costs. However, in our study, baseline CCI scores were higher in the adherent patients, giving credence to the theory that 5-ASA adherence may decrease utilization and costs despite the higher baseline CCI scores. Patients therefore were likely to benefit (i.e., have lower medical resource utilization) from adhering to their medication, regardless of their prior overall medical condition. Our study thus corroborates the findings of Kane[14] that nonadherence leads to higher cost of care. Increased medication costs among the adherent patients were more than offset by significant reductions in other cost sectors, such as inpatient costs, ER admission costs, outpatient physician and other visits, and nonpharmacy visit costs. Our results were consistent with the American Gastroenterological Association’s report on the burden of gastrointestinal diseases, which found pharmacy and inpatient costs to be the key cost driver in the overall burden of UC [2].

While our data source presents unique advantages in assessing medication adherence and associated outcomes, our study is subject to several limitations inherent in most analyses of retrospective claims data. Therapy may be interrupted for clinically appropriate reasons, such as side effects associated with 5-ASAs and abnormal laboratory results. If this occurred, the calculated MPR would underestimate actual adherence rates. We also assumed complete medication ingestion, but patients may have disposed of medication prior to refill or stockpiled medication for future use [29]. This would cause the MPR to overestimate actual adherence. Finally, some of the defined UC-related prescriptions could have been used for other indications. Despite these limitations, administrative claims data remain a reliable and well-accepted source for estimating adherence with chronic-use medications by using validated measures, such as the MPR [18,19,29,30]. Second, all UC patients were identified as such through the analysis of ICD-9-CM diagnosis codes that, if recorded inaccurately, may have caused some patients to be misclassified into the defined groups. The accuracy of our results therefore depends on the accuracy of the data recorded in the LifeLink database. The impact of misclassification bias stemming from analyses of claims data has been described in previous research [31,32]. Third, in order to select patients with active UC, we required that patients have least one corticosteroid prescription in the 12-month period following 5-ASA initiation. A possible confounder is that some patients might have stopped their 5-ASA therapy due to non-response or because they are more severe patients and moved on to corticosteroids, thereby appearing to be nonadherent to 5-ASAs, not because of nonadherent medication-taking behavior but for a clinically appropriate reason. If this hypothesis was true, we would expect corticosteroid use to be greater in the non-adherent group. We therefore examined the total number of prescriptions for corticosteroids over the 12-month follow-up period for the adherent and non-adherent groups and found the reverse to be true: the adherent group had more steroid prescriptions on average (4.1) compared to the non-adherent group (3.4), and stayed on steroids for a longer duration (88.6 vs. 67.9 days). This demonstrates that these patients are more adherent in general and are therefore more adherent to their corticosteroid prescriptions too. Therefore, it seems evident that higher severity, as measured by theuse of corticosteroids in the non-adherent group is not an explanation for lower 5-ASA adherence in that group. Fourth, we did not have any data on important clinical factors such as treatment-related adverse events or disease severity, or demographic factors such as race and socioeconomic status, any of which could impact both medication adherence and health care resource utilization. We did however find no significant difference in the proportion of patients using other 5-ASA drugs such as immunosuppressants and antibiotics between the two study groups, indicating that the groups had similar disease severity. Fifth, our study did not control for pre-index resource utilization. It is possible that nonadherent patients had greater costs prior to 5-ASA initiation. Finally, our study focused only on direct all-cause costs to commercial third-party payers, and is not representative of other health care payers such as Medicare or Medicaid. Also, there are other indirect costs, such as lost wages and reduced workplace productivity, which must be assessed in order to gain an understanding of the complete societal impact of UC.

## Conclusions

In conclusion, this study finds adherence to oral 5-ASAs to be suboptimal and that patients who are nonadherent to therapy have greater health care costs. Efforts to promote oral 5-ASA adherence in patients with UC may lead to cost-savings for third-party payers and other stakeholders.

## Endnotes

^a^At the request of the health services with which we worked, we have been asked to use this term to refer to our informants in order to ensure anonymity.

## Competing interests

Debanjali Mitra and Keith L. Davis are employees of RTI Health Solutions, an independent contract research organization that received research funding from Shire for this study. Dr. Paul Hodgkins and Linnette Yen are employees of Shire and own stock in the company. Dr. Russell D. Cohen is employed at the University of Chicago Medical Center and serves as a consultant to Shire. The author(s) declare that they have no other competing interests.

## Authors’ contributions

Debanjali Mitra, Dr. Paul Hodgkins, and Linnette Yen were the primary developers of the study design and contributed to analysis and interpretation of results as well to the development of this manuscript. As principal investigator, Debanjali Mitra had full access to all the data in the study and takes responsibility for the integrity of the data and the accuracy of the data analysis. Debanjali Mitra and Keith L. Davis led all statistical analyses. Debanjali Mitra also served as the primary writer in drafting the manuscript text and in interpreting the results. Dr. Russell D. Cohen provided medical and clinical advice to refine and validate the study, and he participated in the drafting of the manuscript. All authors read and approved the final manuscript.

## Pre-publication history

The pre-publication history for this paper can be accessed here:

http://www.biomedcentral.com/1471-230X/12/132/prepub
